# Monoterpene Thiols: Synthesis and Modifications for Obtaining Biologically Active Substances

**DOI:** 10.3390/ijms242115884

**Published:** 2023-11-01

**Authors:** Denis V. Sudarikov, Liliya E. Nikitina, Patrick Rollin, Evgeniy S. Izmest’ev, Svetlana A. Rubtsova

**Affiliations:** 1 Institute of Chemistry, Federal Research Center “Komi Scientific Center”, Ural Branch, Russian Academy of Sciences, 167000 Syktyvkar, Russia; evgeniyizmestev@rambler.ru (E.S.I.); rubtsova-sa@chemi.komisc.ru (S.A.R.); 2 General and Organic Chemistry Department, Kazan State Medical University, 49 Butlerov St., 420012 Kazan, Russia; nikitl@mail.ru; 3 Institute of Organic and Analytical Chemistry (ICOA), Université d’Orléans et the French National Center for Scientific Research (CNRS), UMR 7311, BP 6759, F-45067 Orléans, France; patrick.rollin@univ-orleans.fr

**Keywords:** monoterpenoids, thiols, asymmetric synthesis, disulfides, thiosulfonates, sulfenimines, sulfinamides, antimicrobial activity

## Abstract

Monoterpene thiols are one of the classes of natural flavors that impart the smell of citrus fruits, grape must and wine, black currants, and guava and are used as flavoring agents in the food and perfume industries. Synthetic monoterpene thiols have found an application in asymmetric synthesis as chiral auxiliaries, derivatizing agents, and ligands for metal complex catalysis and organocatalysts. Since monoterpenes and monoterpenoids are a renewable source, there are emerging trends to use monoterpene thiols as monomers for producing new types of green polymers. Monoterpene thioderivatives are also known to possess antioxidant, anticoagulant, antifungal, and antibacterial activity. The current review covers methods for the synthesis of acyclic, mono-, and bicyclic monoterpene thiols, as well as some investigations related to their usage for the preparation of the compounds with antimicrobial properties.

## 1. Introduction

Sulfur-containing monoterpenoids, and especially thiols, being natural flavoring agents that impart the pleasant aroma to citrus fruits, wine, and black currants, are of interest in the exploration of flavors and fragrances [1,2,3,4]. Synthetic monoterpene thiols, known for their natural enantiomeric purity, have found applications in asymmetric synthesis. For example, pinane, menthane, and bornane thiols are used as chiral auxiliaries [5,6,7,8,9,10], chiral ligands for metal complex catalysis [11,12,13,14], organocatalysts [15], and chiral resolving agents [16]. Recently, there has been a tendency to exploit monoterpenes—in particular, monoterpene thiols—as monomers for producing green polymers [17,18].

The spread of multidrug-resistant pathogenic microorganisms poses the challenge of searching for new antimicrobials with novel modes of action to which microorganisms have not yet developed resistance [19]. The acquisition of genes encoding efflux systems or enzymes able to hydrolyze antimicrobials, the increased biofilm formation, and the structural changes in target molecules and the cell wall reduce the effectiveness of traditional antibiotics [20].

Among the various classes of molecules which can keep down the growth of pathogenic bacteria and fungi, monoterpene derivatives stand out for their broad spectrum of antimicrobial activity [21,22,23]. The ability of monoterpenoids to inhibit the growth of diverse bacteria and fungi has been reported [21,24,25,26,27,28,29].

The combination of terpenes with known antimicrobials increases the activity of the latter [30,31,32]. The introduction of sulfur functional groups into the structure of biologically active terpenes often enhances the antibacterial and antifungal activity of the resulting thio-modified monoterpenoids compared to the original terpenes [21,29,33,34,35,36]. Pinane and menthane sulfides containing a fragment of 2-mercaptoacetic acid methyl ester showed a wide range of antifungal activity against pathogenic strains of *Candida albicans* and a number of mycelial fungi [21,29].

The reason for these synergistic effects may be explained by the increased affinity of terpenes for the membrane or membrane-associated proteins. The binding site for cyclic hydrocarbons, including terpenes, is known to be in the cell membrane of pathogenic microorganisms [37]. Some terpenes, such as limonene, α- and β-pinenes, and γ-terpinene, can suppress respiration and other energy-dependent processes localized in the cell membranes of fungi and bacteria [22,38,39,40,41]. Furthermore, some terpene derivatives interact with eukaryotic cell membranes [29,42].

Only a few reviews have been devoted to the synthesis and biological activity of thio-modified monoterpenoids [21,29,43]. The current review covers methods for the synthesis of acyclic, mono-, and bicyclic monoterpene thiols, as well as some investigations related to their usage for preparing new compounds with antimicrobial properties.

## 2. Synthesis of Monoterpene Thiols

Thiols are one of the most convenient synthons in the synthesis of organosulfur compounds. The typical methods to prepare monoterpene thiols include the electrophilic addition of H_2_S or dithiols to the double bond of monoterpenes; nucleophilic substitution of halides; tosylates/mesylates obtained from corresponding monoterpene alcohols; thia-Michael addition of S-nucleophiles to α,β-unsaturated ketones; nucleophilic epoxide ring opening; nucleophilic substitution of the activated methylene protons; and reduction of sulfochlorides, dithiolanes, thiiranes, and sultones.

### 2.1. Synthesis from Alkenes

**Scheme 1:**
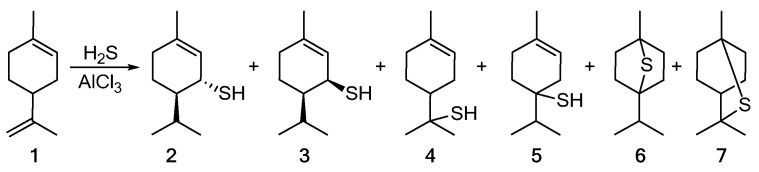
The addition of H_2_S to limonene **1** catalyzed by AlCl_3_.

The synthesis of terpene thiols from limonene, α-pinene, α-, γ-terpinenes, terpinolene, and 3-carene via a reaction of them with H_2_S in the presence of Lewis acids such as AlCl_3_ or AlBr_3_ is described in [44]. The addition of H_2_S usually occurs without selectivity and is accompanied by numerous side reactions, including the rearrangement of the terpene skeleton, especially in cases with bicyclic systems. The addition of H_2_S to limonene **1** catalyzed by AlCl_3_ proceeds with no regioselectivity and gives thiols **2**–**5** in low yields, with the intramolecular cyclization of thiols **4** and **5** at the double bond affording sulfides **6** and **7** as the main products (Scheme 1) [45,46,47].

The interaction of α-pinene **8** with H_2_S under the same conditions leads to products **2**–**7**, as well as cyclic sulfide **9** [44].


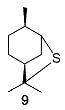


**Scheme 2:**
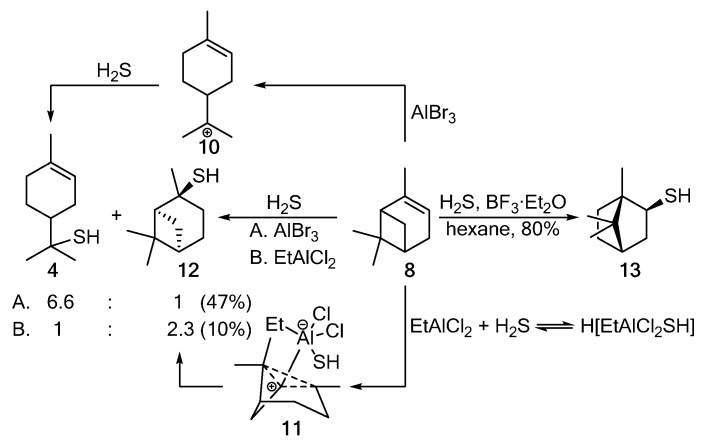
The addition of H_2_S to α-pinene **8**.

Electrophilic thiylation of α-pinene **8** with H_2_S in the presence of AlBr_3_ (A) is followed by the pinene–menthane rearrangement, providing carbocation **10**, which, when reacting with H_2_S, gives thiol **4**. The softer Lewis acid EtAlCl_2_ (B) stereoselectively catalyzes the anti-addition of H_2_S via the formation of intermediate **11** and leads to *trans*-pinane-2-thiol **12** (Scheme 2) [4]. With a strong Lewis acid (BF_3_·Et_2_O) used as a catalyst, the Wagner–Meerwein rearrangement occurs to yield isobornanethiol **13** [4,46]. 

**Scheme 3:**
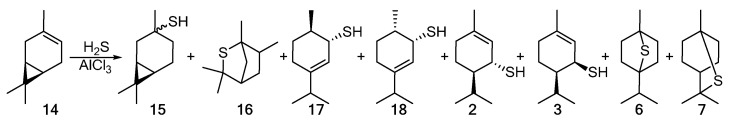
The addition of H_2_S to 3-carene **14** catalyzed by AlCl_3_.

The addition of hydrogen sulfide to 3-carene **14** in the presence of AlCl_3_ proceeds nonselectively to give the products in low yields. The detected products included a mixture of *cis*- and *trans*-thiols **15**; episulfides **16**, **6**, and **7**; and *para*-menthane thiols **17**, **18**, **2**, and **3** (Scheme 3) [44]. 

**Scheme 4:**
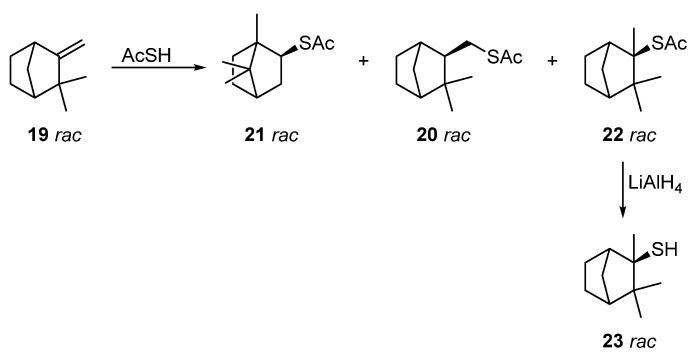
Synthesis of camphane thiol **23**.

Reactions of racemic camphene **19** with thioacetic acid under various conditions were investigated in [48] (Scheme 4). It was established that, under catalyst-free conditions and with a long reaction time (12 h), the anti-Markovnikov product **20** was predominantly formed. The use of *p*-toluenesulfonic acid as a catalyst also leads to thioester **20**, but in a 15% yield. Catalysis with trifluoromethanesulfonic acid (TfOH) and InCl_3_ at different temperatures gives different ratios of products. The optimal yield of thioacetate **21** (75%), a product of the Wagner–Meerwein rearrangement, was achieved using a catalyst TfOH at 40 °C for 20 min. The yield of a by-product, thioacetate **20**, from this procedure does not exceed 25%. The best method to obtain Markovnikov product **22** (82%) with a preserving camphane structure was catalysis via In(OTf)_3_ at ≤0 °C. The deacylation of thioacetate **22** with LiAlH_4_ leads to racemic camphane thiol **23** at an 86% yield.

**Scheme 5:**
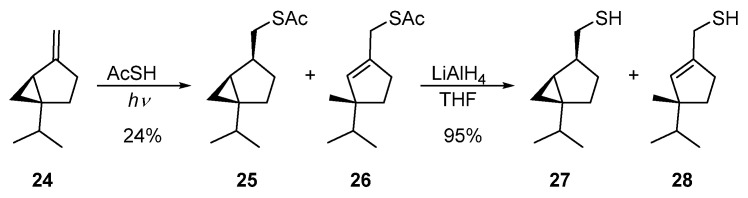
Synthesis of thiols from sabinene **24**.

Photochemical addition of thioacetic acid to (−)-sabinene **24** gives a mixture of anti-Markovnikov bicyclic thioacetate **25** and unsaturated thioacetate **26** in an overall yield of 24% and a 3:1 ratio, respectively [49]. The unexpected formation of thioacetate **26** results from cyclopropane ring cleavage. The mixture of thioacetates **25** and **26** was treated with LiAlH_4_ to produce thiols **27** and **28** in an overall yield of 95% (Scheme 5). The obtained thiols were isolated by preparative capillary GC.

### 2.2. Ene Reaction of Monoterpenes with N-sulfinylbenzenesulfonamide

**Scheme 6:**
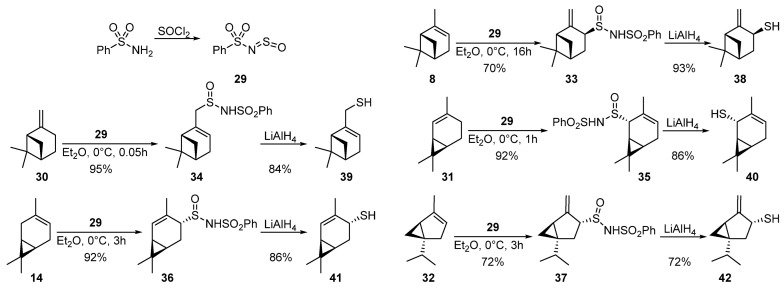
Synthesis of allylic terpene thiols **38**–**42**.

An efficient method for the synthesis of monoterpene allyl thiols using *N*-sulfinylbenzenesulfonamide **29** as an enophile in ene reaction was proposed in the paper [50] (Scheme 6). The interaction of terpenes (α- and β-pinenes **8** and **30**; 2- and 3-carenes **31** and **14**; and α-thujene **32**) with *N*-sulfinylbenzenesulfonamide **29** proceeds at a double bond with the formation of adducts **33**–**37** with a migration of the double bond to an α-position. It should be noted that these reactions occur stereo- and regioselectively. The adducts **33**–**37**, when reduced with LiAlH_4_, provide the corresponding allyl thiols, **38**–**42**.

### 2.3. Synthesis from α,β-Unsaturated Carbonyl Compounds

Thiols are good nucleophiles for thia-Michael addition to α,β-unsaturated carbonyl compounds [51]. However, harsh reaction conditions are required to convert the newly formed sulfide group into a synthetically more versatile SH group. Thioacids (RCOSH) are more attractive as nucleophiles for the Michael addition reaction, since the resulting thioesters can be easily transformed into corresponding thiols under mild conditions [5,52,53].

**Scheme 7:**
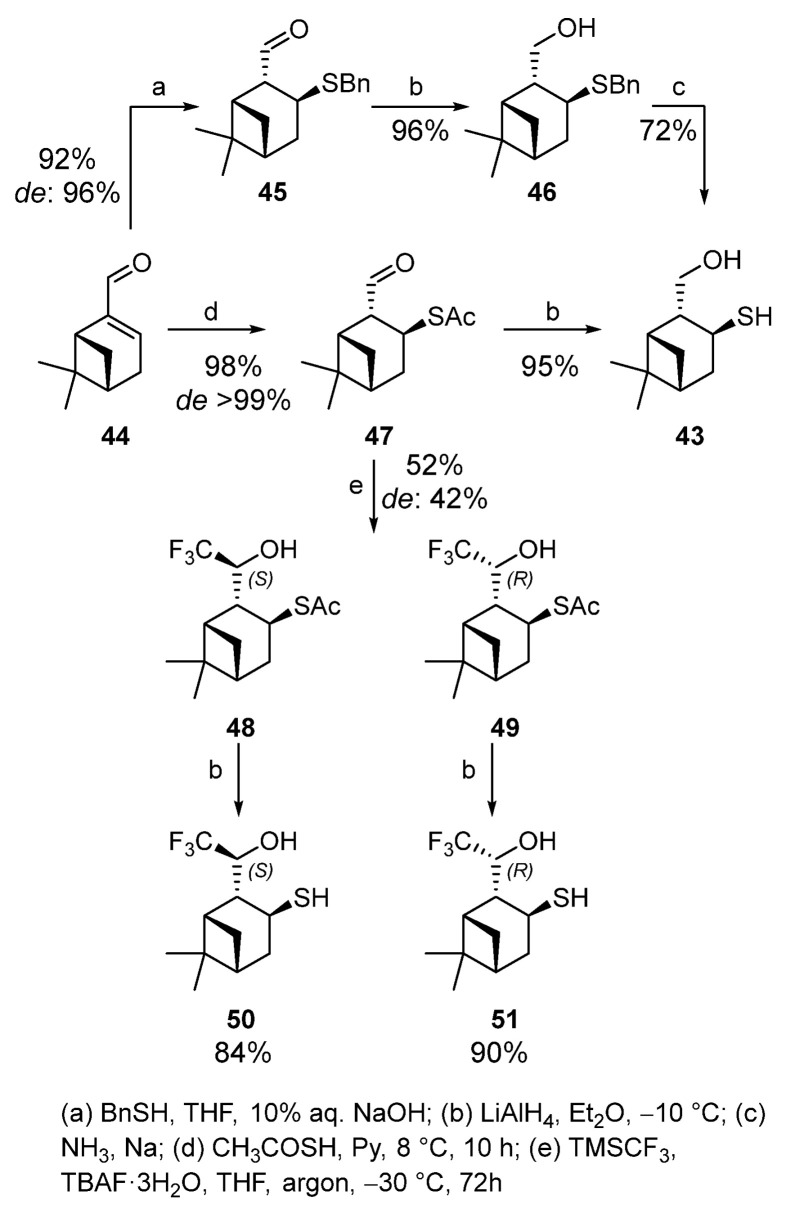
Synthesis of pinane hydroxythiols based on myrtenal **44**.

Myrtenal-based hydroxythiol **43** was synthesized by two methods with a high yield and stereoselectivity [5]. The treatment of (−)-myrtenal **44** with benzylthiol and 10% aqueous NaOH in THF at room temperature for 18 h led to sulfide **45** (yield 92%, *de* 96%). Compound **45** was reduced to the corresponding alcohol **46** (yield 96%) with LiAlH_4_ in Et_2_O, which was then hydrogenolyzed to hydroxythiol **43** under Birch reduction conditions (Scheme 7). The hydrogenolysis did not provide satisfactory results because small differences in reaction conditions altered the reaction course dramatically, sometimes producing a complex mixture of unidentified compounds. The same reaction conditions become reproducible in switching to thioacetic acid as a nucleophilic reagent, which demonstrated a high selectivity when added to (−)-myrtenal **44** to give thioacetate **47** (1,4-addition) in yield of 98% and *de* > 99%. Thioester **47** was reduced by LiAlH_4_ to obtain hydroxythiol **43** in a 95% yield. This one-pot method allowed us to simultaneously convert thioether and aldehyde group to the corresponding thiol and primary alcohol (Scheme 7).

Trifluoromethylation of 2-formylisopinocampheyl-3-thioacetate **47** by Ruppert–Prakash reagent in the presence of tetra-*n*-butylammonium fluoride (TBAF) was carried out at −30 °C for 3 days. Diastereomers **48** and **49** are formed in a 52% total yield and *de* 42% with the predominance of thioacetate **48**. Deacylation of thioacetates **48** and **49** with LiAlH_4_ in dry Et_2_O under an argon atmosphere gives the corresponding thiols **50** and **51** with 84 and 90% yields, respectively (Scheme 7) [54].

**Scheme 8:**
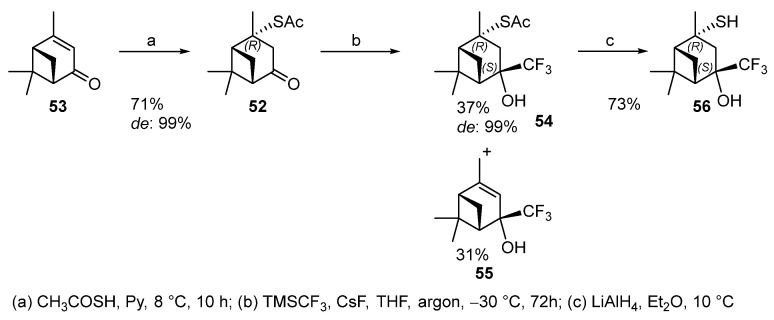
Synthesis of pinane hydroxythiols based on verbenone **53**.

Thioacetate **52** was obtained from (1*S*)-(−)-verbenone **53** by using a procedure similar to the synthesis of 2-formylisopinocampheyl-3-thioacetate **47**. The reaction produces one of two theoretically possible diastereomers with the *R*-configuration of C-2 with a 71% yield (Scheme 8). Thioacetate **52** does not react with the Rupert–Prakash reagent under the above conditions, possibly because of the bulky TBAF use.

The addition of fluorine-containing initiator CsF made it possible to obtain the only (4*S*)-diastereomer **54** in a 37% yield together with trifluoromethyl alcohol **55** (31%) that is a by-product of desulfurization (Scheme 8). Deacylation of thioacetate **54** gave hydroxythiol **56** in 73% yield [54].

**Scheme 9:**
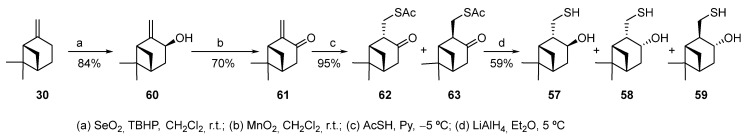
Synthesis of pinane hydroxythiols based on β-pinene **30**.

The synthesis of isomeric hydroxythiols **57**–**59** was carried out on the basis of β-pinene **30** (Scheme 9) [55]. *Trans*-pinocarveol **60** was synthesized via the oxidation of β-pinene **30** with the SeO_2_/TBHP system, and its further oxidation with MnO_2_ led to pinocarvone **61**. An inseparable mixture of two isomeric ketothioacetates (2*S*)-**62** and (2*R*)-**63** in a 2:1 ratio in 95% yield is formed during the thia-Michael reaction of pinocarvone **61** with AcSH in the presence of catalytic amount of pyridine at −5 °C. The reduction of thioacetates with LiAlH_4_ leads to three isomeric hydroxythiols, **57**–**59**.

**Scheme 10:**
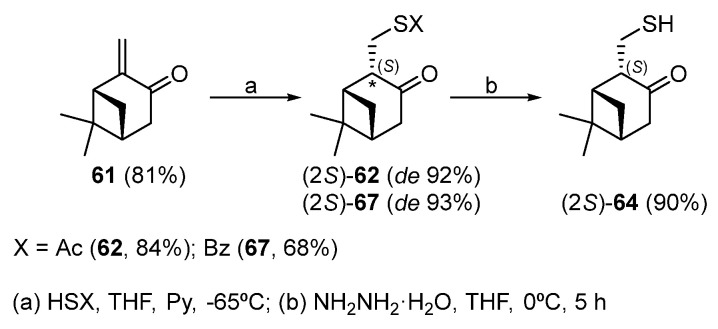
Synthesis of β-ketothiol from pinocarvone **61**.

The synthesis of pinane ketothiols **64** and **65** was implemented from α,β-unsaturated pinane ketones **61** and **66** [56]. To obtain thioacetate **62** from enone **61**, the synthetical protocol proposed in [5] was used. However, the diastereoselectivity of this reaction under the described conditions did not exceed 33%, as mentioned in [55]. The *de* value of thioacetate **62** can be increased from 33 up to 92% if the reaction between pinocarvone **61** and AcSH is carried out in THF in a temperature range from −60 to −65 °C, with pyridine as a co-solvent. The same conditions are applicable for the addition of BzSH to ketone **61**, with thioacetate **67** being formed in this case with a comparable *de* of 93% (Scheme 10). Reducing thioacetate **62** via NH_2_NH_2_·H_2_O affords thiol **64** within 4-5 h in up to a 90% yield, while deacylation of thiobenzoate **67** by the same reagent gives the thiol in only a 38-50% yield due to incomplete conversion. Thus, at comparable maximum *de* values of thioesters **62** and **67**, the preparation of thiol **64** from compound **62** is more optimal, taking into account the higher total yield of thiol and the diacylation time.

**Scheme 11:**
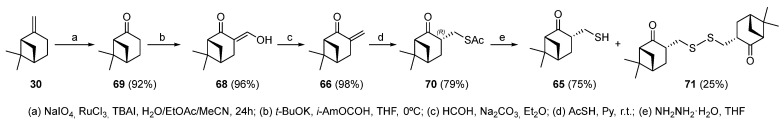
Synthesis of β-ketothiol based on 2-norpinanone **66**.

A multistep synthesis of 2-norpinanone **66** from (−)-β-pinene **30** was provided in [57] (Scheme 11). This compound was obtained via nopinone **69** and then ketoenol **68** formation. Ketoenol **68** was produced in a 96% yield from ketone **69** by its reaction with isoamyl formate and *t*-BuOK in THF at 0 °C for 6 h [56]. The following dihydroxylation of ketoalcohol **68** by formaldehyde in sodium carbonate solution afforded 2-norpinanone **66 [56]**. An addition of thioacetic acid to 2-norpinanone **66** was, for the first time, implemented according to the procedure [5] and then by using pyridine as a catalyst [51] in THF at room temperature [56]. The main product of this reaction was the isomer (3*R*)-**70** (*de* 98%) (Scheme 11). Its deacylation by hydrazine hydrate (NH_2_NH_2_·H_2_O) led to 2-ketothiol **65** and disulfide **71** in a 3:1 ratio, respectively. Because of the mild reducing properties of NH_2_NH_2_·H_2_O and its inability to donate protons, the diacylation proceeds chemoselectively with the preservation of the carbonyl group [58], a behavior that is not typical for LiAlH_4_ when used [55].

**Scheme 12:**
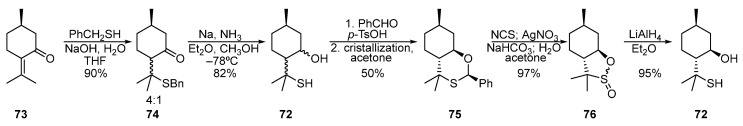
Synthesis of β-hydroxythiol based on pulegone **73**.

Pulegone **73** was used to synthesize *para*-menthane-derived β-hydroxythiol **72** (Scheme 12) [59,60,61,62]. The 1,4-addition of sodium benzyl thiolate to pulegone led to a diastereomeric mixture of ketosulfides **74** in a 4:1 ratio. Then, the mixture **74** was reduced under Birch conditions by Na in liquid NH_3_ to give a mixture of hydroxythiols **72**. Condensation of **72** with benzaldehyde and subsequent crystallization from acetone afforded diastereomerically pure oxathiane **75** in a 50% yield. When oxidized by AgNO_3_ in the presence of NCS, oxathiane **75** is transformed into sultines **76**, the reduction of which with LiAlH_4_ gives pure β-hydroxythiol **72**.

**Scheme 13:**
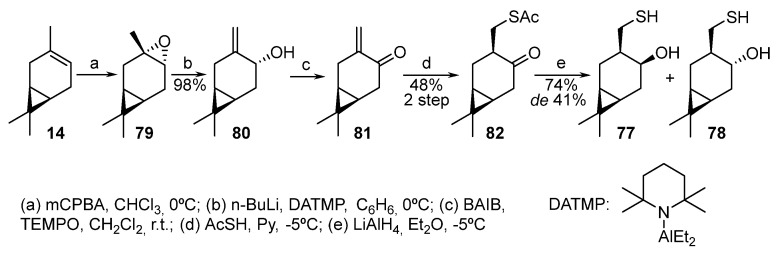
Synthesis of monoterpene hydroxythiols based on 3-carene **14**.

Isomeric α,β-hydroxythiols **77** and **78** were obtained from natural 3-carene **14** (Scheme 13) [63]. 3-Carene, when oxidized by *m*-CPBA, selectively forms *trans*-epoxide **79**, which is isomerized in the presence of diethylaluminum 2,2,6-tetramethylpiperidide (DATMP) to enol **80** [64]. The oxidation of alcohol **80** to enone **81** is successfully implemented by the bis(acetoxy)iodobenzene (BAIB)–2,2,6,6-tetramethylpiperidine 1-oxyl (TEMPO) system. Enone **81**, being an unstable compound, cannot be isolated in its pure form. The two-step thia-Michael addition of AcSH to α,β-unsaturated ketone **81** proceeds in one pot in pyridine. As a result, only one of the two theoretically possible diastereomers, thioacetate **82**, is formed. The subsequent reduction of ketothioacetate **82** by LiAlH_4_ leads to two diastereomeric β-hydroxythiols, **77** and **78**, in a 1:2 ratio, respectively [63].

### 2.4. Synthesis from Alcohol via Tosylates, Halides, Isothiouronium Salts

**Scheme 14:**
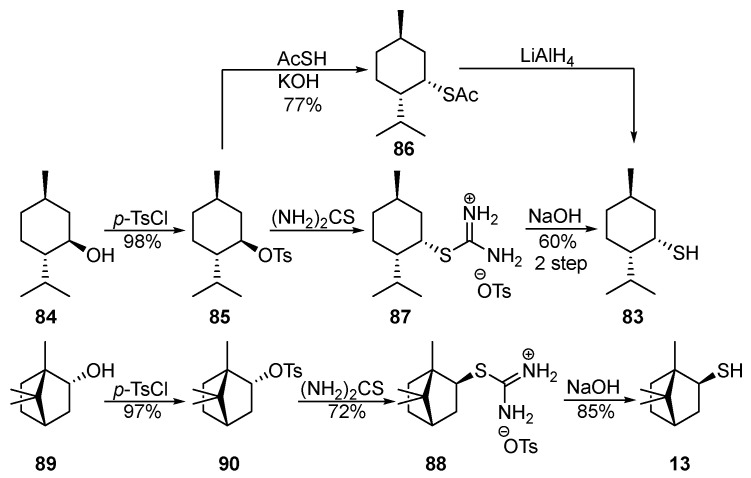
Synthesis of neomenthanethiol **83** and isobornanethiol **13**.

The works [65,66,67,68] cover the methods for the selective preparation of neomenthanethiol **83** using thioacetic acid (AcSH) (Scheme 14). Starting menthol **84** reacts with *p*-TsCl in pyridine to form tosylate **85**, which, when heated with AcSK, gives thioacetate **86** in a 77% yield. Substitution of the OTs (*p*-toluenesulfonate, tosylate) by the AcS-group occurs with an inversion of the chiral center via the S*_N_*2 mechanism. The reduction of **86** by LiAlH_4_ provides diastereomerically pure thiol **83** in a 26–40% yield (Scheme 14).

Neomenthanethiol **83** [68,69] and isobornanethiol **13** [68,70,71,72] were also synthesized in good yields via isothiouronium salts **87** and **88**, proceeding from alcohols **84** and **89** (Scheme 14).

**Scheme 15:**
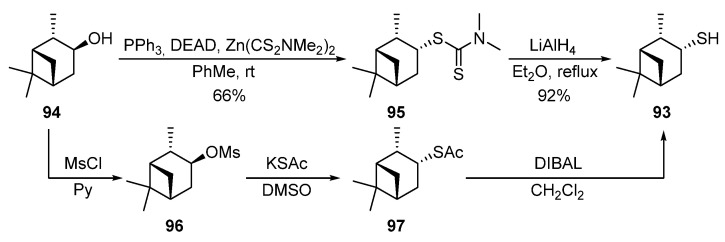
Synthesis of (1*S*,2*S*,3*R*,5*R*)-3-pinanethiol **93**.

In addition to neomenthanethiol **83** and isobornanethiol **13**, the authors of [68] prepared 4-caranethiol **91** and *cis*-myrtanethiol **92** using the same method. 


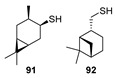


(−)-(3*R*)-Pinanthiol **93** was proposed to be obtained via the Mitsunobu-type procedure from (+)-isopinocampheol **94** [6,7] (Scheme 15). The reaction of the alcohol **94** with zinc *N*,*N*-dimethyldithiocarbamate in the presence of triphenylphosphine and diethylazodicarboxylate (DEAD) is accompanied by an inversion of C-3 configuration and leads to dithiocarbamate **95** in a 66% yield. Dithiocarbamates baced on menthol **84** and borneol **89** were also obtained by the same procedure [73,74]. The reduction of dithiocarbamate **95** by LiAlH_4_ gives thiol **93** in a 92% yield. The approach to obtain thiol **93** through the corresponding mesylate **96** and thioacetate **97** was described in [12].

**Scheme 16:**
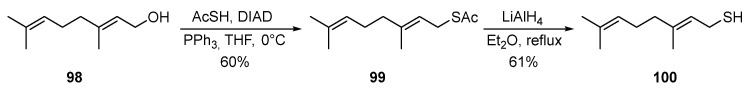
Synthesis of thiogeraniol **100**.

Geraniol **98** reacts with thioacetic acid under Mitsunobu-type conditions [75] to form thioacetate **99** in a good yield, which, when treated with LiAlH_4_, is converted into the corresponding thiol **100** in a 61% yield (Scheme 16) [76].

**Scheme 17:**
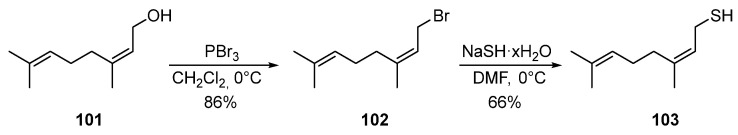
Synthesis of thionerol **103**.

The ability of nerol **101** to be converted into bromide **102** under the action of PBr_3_, and then into thiol **103** by using NaSH via two successive nucleophilic substitutions with yields of 86 and 66%, respectively, was described in [77] (Scheme 17).

**Scheme 18:**
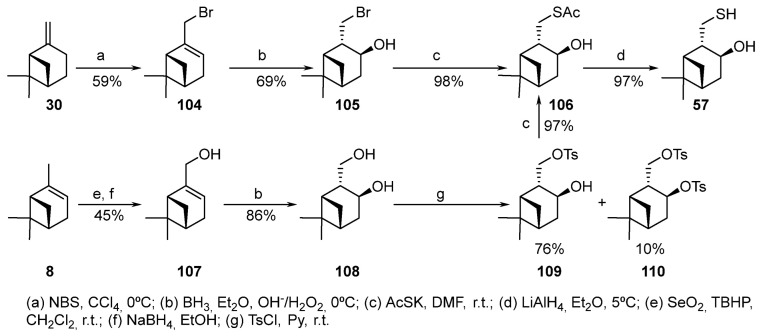
Synthesis of 10-hydroxyisopinocampheylthiol **57** from α- and β-pinene.

Diastereomerically pure hydroxythiol **57** can also be obtained via two alternative routes [55]. The first one involves the bromination of β-pinene **30** by NBS (*N*-bromosuccinimide) to form myrtenyl bromide **104**, which undergoes hydroboration–oxidation and is selectively transformed to bromoalcohol **105.** The nucleophilic replacement of bromide by thioacetate AcS^−^ leads to compound **106**, which can also be synthesized starting from α-pinene **8** (Scheme 18). The second route is associated with the oxidation of α-pinene **8** to myrtenal, followed by its reduction to myrtenol **107**, which is converted into diol **108** by the same hydroboration–oxidation procedure. The further reaction of tosyl chloride with diol **108** leads to both monotosylate **109** (76%) and ditosylate **110** (10%). The nucleophilic substitution of the *para*-toluenesulfonate group in **109** by AcS^−^ also results in thioacetate **106**. When reduced, thioacetate **106** affords hydroxythiol **57** (Scheme 18) [55].

### 2.5. Nucleophilic Substitution of the Activated Methylene Proton

**Scheme 19:**
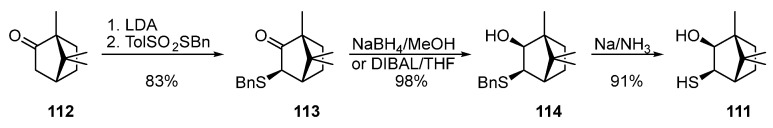
Synthesis of bornane α-hydroxythiol **111** from camphor **112**.

The synthesis of bornane α-hydroxythiol **111** was described in [78,79] (Scheme 19). The nucleophilic substitution of a proton of the activated methylene group in camphor **112** by benzyl *p*-toluenesulfonate promoted by LDA leads to the formation of ketosulfide **113**, which, being reduced by NaBH_4_ in methanol or dibutylaluminum hydride (DIBAL) in THF, gives hydroxysulfide **114**, which is capable of being transformed into hydroxythiol **111** by the Birch reduction. 

### 2.6. Epoxide and Thiiran Ring Opening

**Scheme 20:**
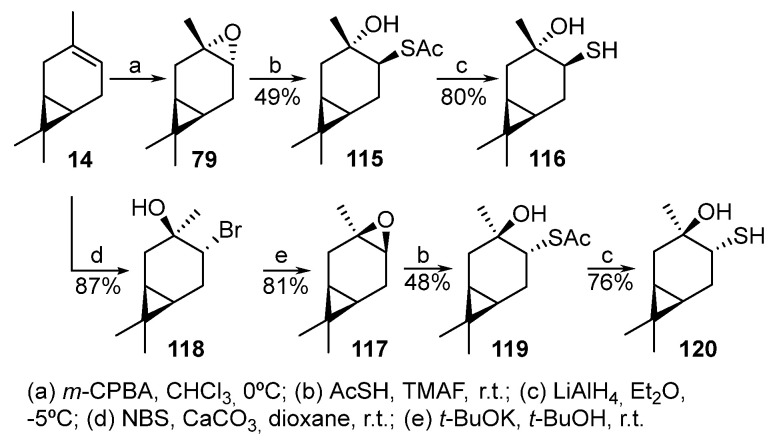
Synthesis of monoterpene hydroxythiols **116** and **120** based on 3-carene **14**.

The nucleophilic ring opening of epoxide **79** with AcSH catalyzed by tetramethylammonium fluoride (TMAF) yields hydroxythioacetate **115**, which is readily deacylated by LiAlH_4_ to form the corresponding α-hydroxythiol **116** (Scheme 20).

*Cis*-epoxide **117** was obtained according to the known method [80] through bromohydrin **118** in 70% total yield. The interaction of epoxide **117** with AcSH in the presence of TMAF leads to thioacetate **119**, the deacylation of which gives α-hydroxythiol **120** (Scheme 20) [63].

**Scheme 21:**
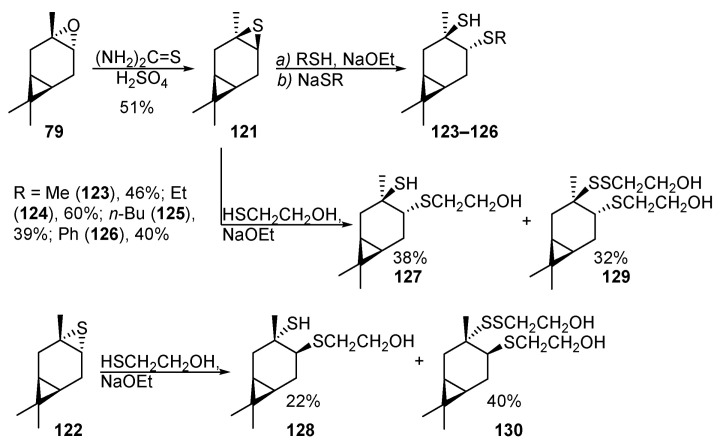
Sulfenylation of carane thiiranes **121** and **122**.

The nucleophilic sulfenylation of carane thiiranes, *cis*-**121** and *trans*-**122**, by mono- (MeSH, EtSH, *n*-BuSH, PhSH) and bifunctional (HSCH_2_CH_2_OH) thiols, promoting with sodium ethoxide and thiolates, affords mercaptosulfides **123–128** with only moderate yields. By-product disulfides **129** and **130** are additionally formed during the reaction of thiiranes **121** and **122** with 2-mercaptoethanol (Scheme 21) [43].

### 2.7. Reduction of Thiiranes, Thiolanes, Sulfonyl Chlorides, and Sultones

**Scheme 22:**
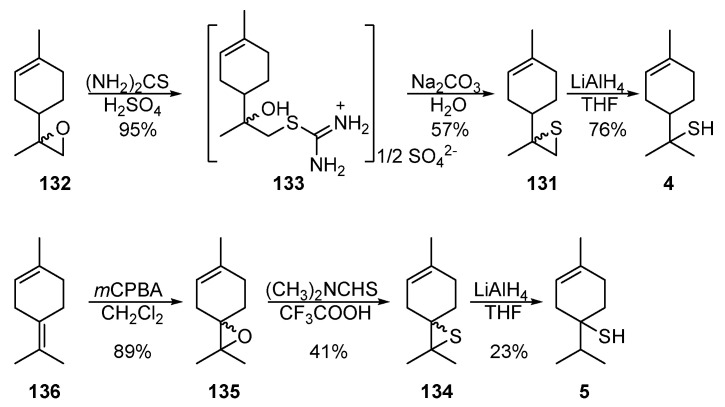
Scheme for the synthesis of racemic 1-*p*-menthene-8-thiol **4** and 1-*p*-menthene-4-thiol **5**.

Monoterpene thiols can be obtained via the reduction of thiiranes. A method for the directed synthesis of racemic thiol **4** from thiirane **131** through oxirane **132** and isothiouronium salt **133** was described in [47]. The sequential reflux of epoxide **132** with thiourea and Na_2_CO_3_ leads to the corresponding thiirane **131**, the reduction of which by LiAlH_4_ gives thiol **4** in a moderate yield. A similar protocol for obtaining racemic thiol **5** was reported in [1]; however, thiiran **134** in this study was synthesized from oxirane **135** using the *N*,*N*-dimethylthioformamide (DMTF)–TFA system as a reagent (Scheme 22).

**Scheme 23:**
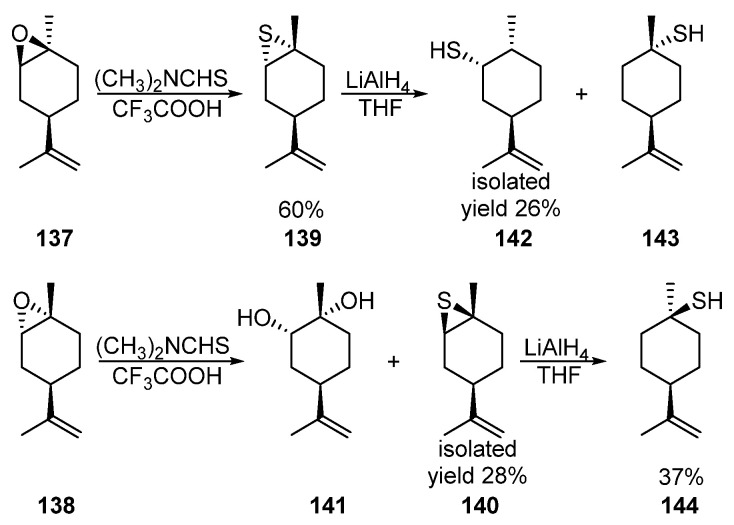
Synthesis and reduction of *para*-menthane thiiranes **139** and **140**.

*Trans*-limonene-1,2-epoxide **137** and *cis*-1,2-limonene-1,2-epoxide **138** were transformed by the DMTF-TFA system into *cis*-**139** and *trans*-1,2-epithio-*p*-ment-8-ene **140**, respectively (Scheme 23) [2]. The yield of thiirane **140** is lower than that of thiirane **139**, since the reaction is accompanied by the formation of the by-product diol **141**, which is yielded during the acid hydrolysis of epoxide **138**. The reductive cleavage of the thiirane ring of **139** proceeds readily to give thiols **142** and **143**, of which only thiol **142** was isolated in its pure form. Thiirane **140** was proposed to reduce to thiol **144** at only a 37% yield.

**Scheme 24:**
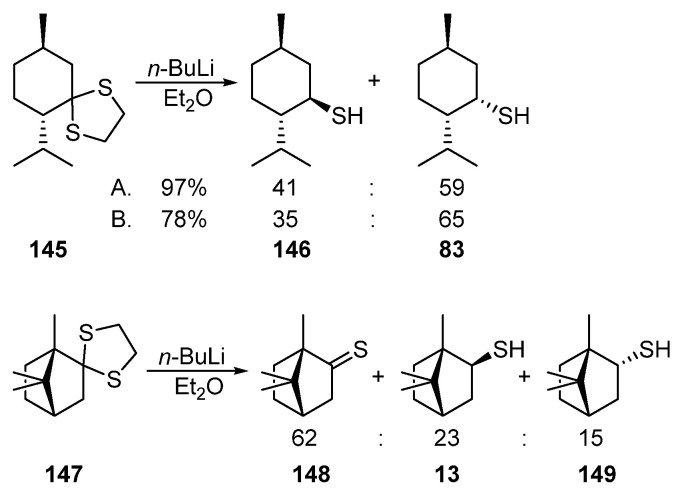
Reductive cleavage of menthone dithiolane **145** and camphor dithiolane **147**.

Thioketals can also be used as the starting compounds for the synthesis of monoterpene thiols. Thus, the reductive cleavage of menthone dithiolane **145** using *n*-BuLi leads to the diastereomeric mixture of menthanethiol **146** and neomenthanethiol **83** (Scheme 24) (A) [81], (B) [82].

The reductive cleavage of camphor dithiolane **147** induced by *n*-BuLi produces thiocamphor **148** (62%) as the major product; the mixture of *exo*-**13** and *endo*-**149** thiols accounts for only 38% (Scheme 24) [83].

**Scheme 25:**
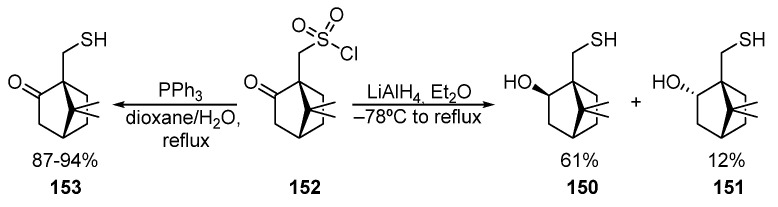
Synthesis of 10-thioisoborneol **150**, 10-thioborneol **151**, and 10-thiocamphor **153**.

Some methods to obtain bornane β-hydroxythiols **150** and **151** by reducing camphor-10-sulfonyl chloride **152** are described in [22,23,24]. As a result of this transformation, two diastereomeric hydroxythiols, **150** and **151**, are formed (Scheme 25). Camphor-10-sulfonyl chloride **152** can also be selectively converted into ketothiol **153** by using PPh_3_ as a reducing agent [84,85].

**Scheme 26:**
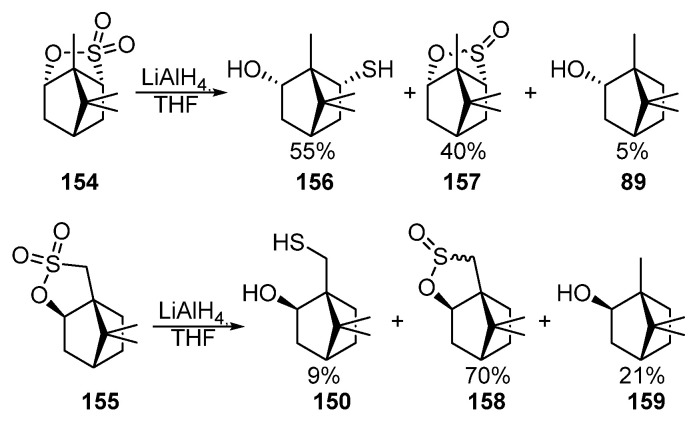
Reduction of monoterpene sultones **154** and **155**.

The authors of [86,87] carried out the reduction of bornane sultones **154** and **155** by LiAlH_4_ in THF to form the corresponding mixture of hydroxythiols **156** and **150**, sultines **157** and **158**, borneol **89**, and isoborneol **159** (Scheme 26).

## 3. Syntheses Involving Monoterpene Thiols for the Production of Biologically Active Substances

**Scheme 27:**
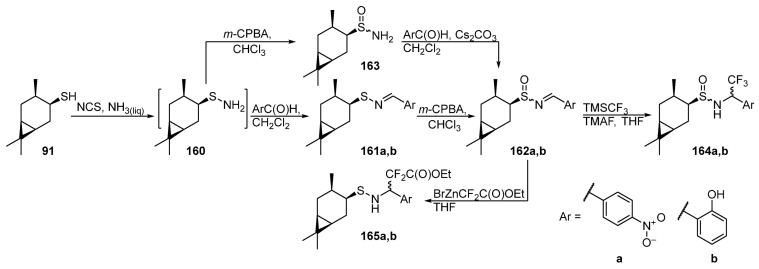
Synthesis of sulfenimines, sulfinamides, sulfinimines, and *N*-substituted sulfinamides based on 4-caranethiol **91**.

CF_3_-Containing *N*-substituted sulfinamides synthesized from 4-caranethiol via sulfenimines and sulfinimines are reported in [34]. 4-Caranethiol **91**, when treated with NCS in liquid ammonia, forms the unstable sulfenamide **160**, which is condensed in situ with 4-nitrobenzaldehyde or salicylic aldehyde to produce sulfenimines **161a,b** in 73-87% yields. The further asymmetric oxidation of sulfenimines by various oxidants and oxidation systems (*m*-CPBA, TBHP, CHP–VO(acac)_2_, and H_2_O_2_–VO(acac)_2_–L*) leads to the corresponding diastereomeric sulfinimines **162a,b** (85–99%). In the work [34], a convenient one-step procedure for the synthesis of chiral primary sulfinamides **163** (overall yield 65%, *de* 12%) from 4-caranethiol **91** via the in situ treatment of sulfenamide **160** with *m*-CPBA was proposed. Sulfinamide **163** also reacts with salicylic- and 4-nitrobenzaldehyde to give sulfinimines **162a,b** in yields from 75 up to 85%. The addition of the Ruppert–Prakash reagent to sulfinimines **162a,b** provides diastereomeric *N*-substituted trifluoromethyl sulfinamides **164a,b** (yield 68-85%). Similarly, using the Reformatsky reagent based on ethyl bromodifluoroacetate allows us to obtain fluorinated *N*-substituted sulfinamides **165a,b** (42–74%, *de* 9-81%) from sulfinimines **162a,b** (Scheme 27). All diastereomers indicated in Scheme 27 were isolated in pure forms by column chromatography and evaluated for antimicrobial activity against ESKAPE pathogens (six highly virulent and antibiotic-resistant bacterial pathogenic bacteria, including *E. faecium*, *S. aureus*, *K. pneumoniae*, *A. baumannii*, *P. aeruginosa*, and *Enterobacter* spp.) [88], fungi *C. Albicans*, and *C. neoformans*. As the reference antimicrobials for Gram-negative and Gram-positive bacteria, colistin and vancomycin were used, respectively, and for the fungi, fluconazole was applied.

Compounds **161a**, (*S*_S_)-**162b**, (*R*_S_*S*)-**164a**, (*S*_S_*R*)-**164b**, (*R*_S_*R*)-**165a**, and (*S*_S_*S*)-**165b** at a concentration of 32 μg/mL showed antibacterial activity against *Acinetobacter baumannii*, and sulfinamide (*R*_S_*R*)-**165a** has antifungal activity against *Candida albicans*. The MIC (minimum inhibitory concentration) value of (*R*_S_*R*)-**165a** against *Candida* was 0.25 μg/mL. This compound also showed moderate cytotoxicity against human embryonic kidney cells (Hek-293) at a concentration of 32 μg/mL. All of this indicates that sulfinamide (*R*_S_*R*)-**165a** is not only a selective antifungal agent but also a promising compound for further medical trials.

**Scheme 28:**
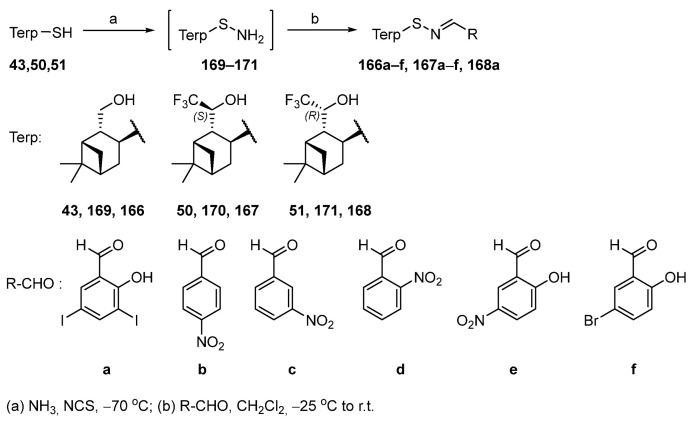
Synthesis of sulfenimines based on pinane hydroxythiols.

A similar approach was used to synthesize sulfenimines **166a**–**f, 167a**–**f,** and **168a** based on trifluoromethylated monoterpene thiols **43, 50,** and **51** [5,54] by varying the stereochemistry of the terpene moiety and the aldehyde structure [35]. The interaction of thiols **43, 50,** and **51** with NCS in liquid ammonia gives sulfenamides **169**–**171**, which can be transformed to sulfenimines **166a**–**f, 167a**–**f,** and **168a** in yields of up to 81% by condensation with various aldehydes (Scheme 28).

The antimicrobial activity of the newly synthesized sulfenimines **166a**–**f, 167a**–**f,** and **168a** was assessed against Gram-positive methicillin-susceptible *S. aureus* (MSSA) and methicillin-resistant *S. aureus* (MRSA), Gram-negative bacterium *P. aeruginosa*, and a fluconazole-sensitive *C. albicans*. These microorganisms are characterized by a high frequency of resistant isolates and cause diseases of various mucous membranes, the skin, and the respiratory tract.

Compounds **166a**, **167a**, **166b**, and **167e** inhibited the growth of all tested pathogens, although the activity was moderate and the MIC values (8–64 µg/mL) were generally higher than those of the reference antimicrobials (amikacin, ampicillin, ciprofloxacin, fluconazole, and benzalkonium chloride). It is important to note that trifluoromethylated sulfenimines with salicylic fragments **168a** and **167f** were active only against methicillin-resistant *S. aureus* and *C. albicans*, and **168a** was even more active than fluconazole (MIC 8 µg/mL). In addition, sulfenimines with a CF_3_ group in the terpene moiety and salicylaldehyde fragment **167a**, **168a**, **167b**, and **167f** exhibit greater antifungal activity (MIC 8–32 µg/mL) in contrast to the non-fluorinated analogues **166a**, **166e**, and **166f** (MIC ≥ 64 µg/mL).

However, most of the synthesized compounds are highly cytotoxic to embryonic bovine lung (EBL) cells. All new compounds have selectivity indices (SI, the ratio of toxicity to MIC) of 2–4, showing their high relative toxicity, which reduces the possibility to further use these compounds as potential antibiotics and indicates the need for further optimization of the structure with reducing the negative effect on eukaryotic cells.

**Scheme 29:**
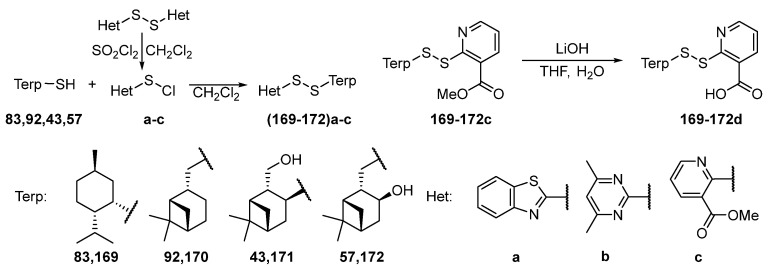
Synthesis of asymmetric monoterpenyl hetaryl disulfides (**169**–**172)a**–**d**.

Some natural [89,90,91,92,93] and synthetic [94,95,96] lipophilic disulfides have antimicrobial properties. In [36], novel unsymmetrical monoterpenylhetaryl disulfides (**169**–**172)a**–**d** based on monoterpene thiols **83**, **92**, **43**, and **57** and heterocyclic disulfides were synthesized in 48–88% yields (Scheme 29). Disulfides **169c**–**172c** with 2-mercaptonicotinic acid methyl ester moiety were converted to the corresponding acids **169d**–**172d** to provide yields of them up to 73–95%. The obtained compounds were evaluated for antibacterial and antifungal activity, cytotoxicity, and mutagenicity. Amikacin and fluconazole were used as antimicrobial references.

Unsymmetrical disulfides **169a**–**d** with a neomentane fragment showed antimicrobial activity against both *S. aureus* strains, with MICs of 16–32 μg/mL. Disulfides **170b** (MIC 16 μg/mL) and **170d** (MIC 16 μg/mL) have the highest activity against the MSSA among the compounds **170a**–**d**. Disulfides **172a**–**c** bearing an OH group at the C-3 position of the terpene fragment did not demonstrate any antibacterial properties. Pinane disulfides **171a**–**c** with a hydroxymethyl group at C-10 in their biological activity turned out to be similar to neomentane thiotherpenoids **169a**–**c** and showed MIC values of 32–64 µg/mL. Only disulfides **169a**–**c**, **170a**, **170b**, and **171a** were capable of inhibiting *Pseudomonas aeruginosa*. Along with that, there were no disulfides among **(169**–**172)a**–**c** with pronounced antifungal activity against the clinical isolate of *C. albicans*. However, disulfides **169**–**172d** containing a 2-mercaptonicotinic acid moiety nevertheless demonstrated antifungal activity (MIC 16–128 μg/mL).

In general, the synthesized asymmetric monoterpenyl hetaryl disulfides (**169**–**172)a**–**d** possess high cytotoxicity (CC_50_) against EBL. Pinane disulfides **170a**, **170b**, and **171d** showed the lowest toxicity. For neomenthane disulfides **169a** and **169b**, mutagenicity was revealed in the Ames test on *Salmonella typhimurium* [97].

**Scheme 30:**
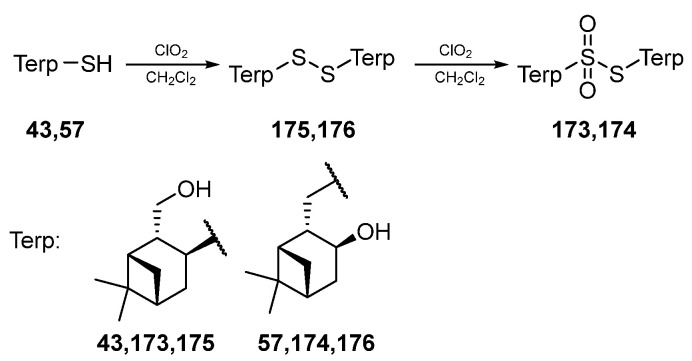
Synthesis of hydroxypinane thiosulfonates **173** and **174**.

Thiosulfonates **173** and **174** were obtained via the oxidation of pinane hydroxythiols **43** and **57** with chlorine dioxide in yields of 46–58% [33] (Scheme 30) and tested for antimicrobial activity against five bacterial strains (*Escherichia coli*, *Klebsiella pneumoniae*, *Acinetobacter baumannii*, *Pseudomonas aeruginosa*, and *Staphylococcus aureus*) and antifungal activity against two fungal strains (*Candida albicans* and *Cryptococcus neoformans*). Colistin and vancomycin were used as reference antibiotics against bacteria, and fluconazole against the fungi. The results showed that sulfonothioates **173** and **174** are active against *Candida albicans*; meanwhile, compound **173** also showed activity against *S. aureus* and *C. neoformans* at 32 μg/mL. 

**Scheme 31:**
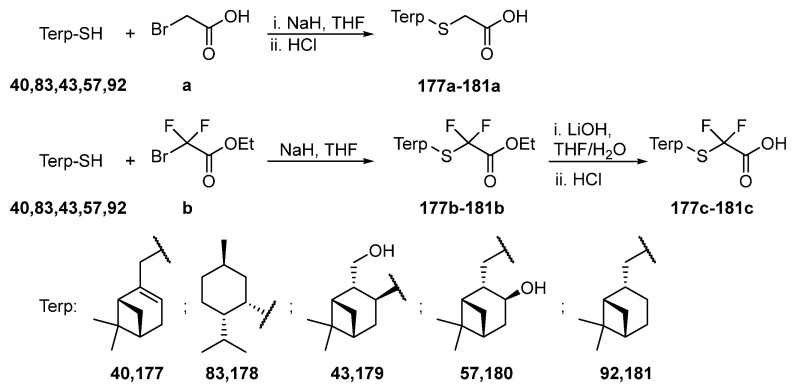
Synthesis thio-monoterpene carboxylic acids **(177**–**178)a–c**.

Thio-modified monoterpene carboxylic acids **177a**–**181a** were produced in 82–98% yields via the reaction of monoterpene thiols such as myrtenethiol **40**, neomenthanethiol **83**, 10-hydroxyisopinocamphenylthiol **43**, 3-*trans*-hydroxy-*cis*-myrtanethiol **57**, and *cis*-myrtanethiol **92** with bromoacetic acid and NaH in THF at 4 °C (Scheme 31) [98]. Similarly, thiols **40**, **83**, **43**, **57**, and **92** react with 2-bromo-2,2-difluoroacetic acid ethyl ester in THF in the presence of NaH. The resulting ethyl esters, i.e., **177b**–**181b**, were not isolated in pure their forms. Thio-monoterpene carboxylic acids **177c**–**181c** were obtained in 56–80% yields by treating the reaction mixture with an aqueous LiOH solution (Scheme 31) [98].

According to the results of antimicrobial activity testing, all compounds except **179c** and **180c** exhibit weak activity against Gram-positive *S. aureus* (MIC 64–128 μg/mL). All compounds, except **178c**, **179c**, and **180c**, are equally active against both MSSA and MRSA. Difluoroacetic acid derivatives **177c**–**181c** have reduced antibacterial activity compared to their non-fluorinated analogues **177a**–**181a**. The acid with a neomenthane moiety **178a** showed weak antifungal activity against *C. albicans* (MIC 128 μg/mL), which is resistant to fluconazole. Ampicillin, amikacin, benzalkonium chloride, and fluconazole were used as reference standards.

## 4. Application of Monoterpene Thiols in Asymmetric Synthesis

Monoterpene thiols, known for their natural enantiomeric purity, have found applications in asymmetric synthesis. To reveal the synthetic potential of monoterpene thiols, we provide some examples of their application in asymmetric synthesis.

**Scheme 32:**
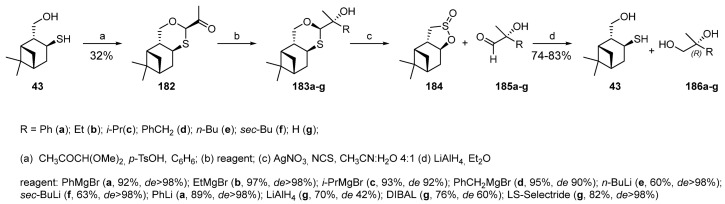
Synthesis of chiral diols **186a–g** using hydroxythiol **43** as a chiral auxiliary.

As an example, the work of [5] can be given, which covers a method of using pinane hydroxythiol **43** as a chiral auxiliary to synthesize certain chiral aldols and diols (Scheme 32). When thiol **43** was treated with α,α-dimethoxyacetone, a single diastereomer, ketooxathiane **182**, was formed with a yield of 32%. The further addition of Grignard reagents and organolithium compounds at the C=O of **182** afforded the corresponding alcohols **183a–g** in good yields and high diastereoselectivity. The configuration of a newly formed chiral center of the major diastereomers was assigned as *R* for tertiary alcohols **183a–f** and *S* for a secondary one, **183g**, due to the change in seniority of substituents. LS-Selectride (lithium trisiamylborohydride) reduces ketone **182** more selectively than LiAlH_4_ and DIBAL (diisobutylaluminum hydride). Compounds **183a–g** reacted with AgNO_3_ and NCS by opening the oxothiane ring to give sultine **184** and aldols **185a–g**, which are not isolated in an individual form. By reducing with LiAlH_4_, this mixture was converted into the separable non-racemic thiol **43** (63–72%) and diols **186a–g** in a yield of 74 up to 83%.

The similar approaches using hydroxythiol **43** for the preparation of chiral diols, as well as α-hydroxy acids, are also described in [99,100,101].

**Scheme 33:**
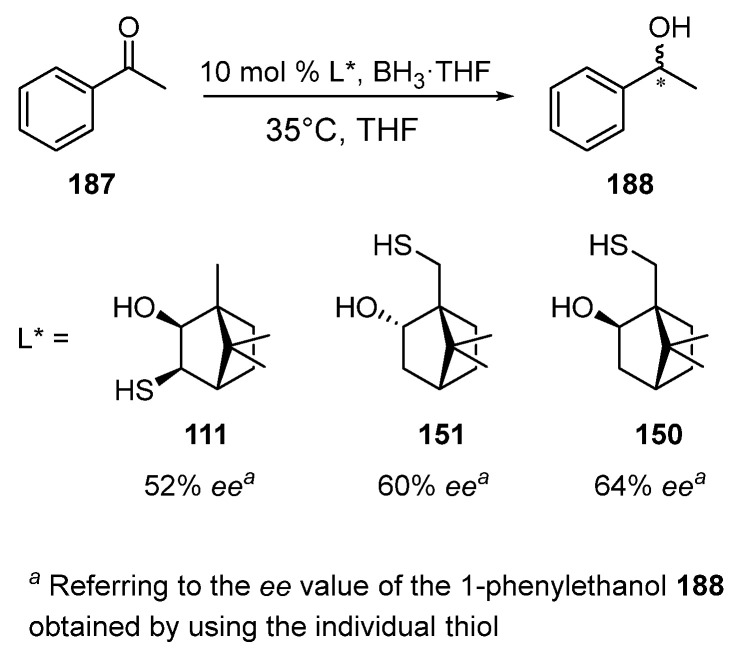
Synthesis of chiral 1-phenylethanol **188** using hydroxythiols **111, 151**, and **150** as chiral organocatalysts.

Chiral bornane 1,2- and 1,3-hydroxythiols **111**, **150**, and **151** were evaluated as catalysts for the asymmetric reduction of prochiral ketones with borane (Scheme 33) [14,15]. Thus, acetophenone **187** was reduced to 1-phenylethanol in yields greater than 90% and in good enantioselectivity. The solvent nature did not affect the reaction enantioselectivity, and the stoichiometric ratio of catalyst to substrate used slightly increased it to 75%. In another work [102], a 96% yield and 87% *ee* were achieved for alcohol **188** by replacing the boron hydrogenating agent with borane dimethyl sulfide, conducting the reaction in toluene at 50 °C with hydroxythiol **111** as an organocatalyst.

**Scheme 34:**
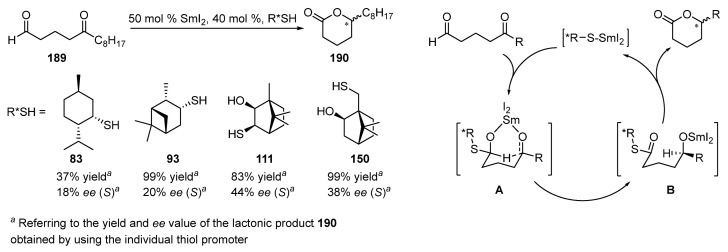
Enantioselective formation of δ-lactone **190** via the treatment of **189** with SmI_2_ and chiral thiols **83**, **93**, **111**, and **150**.

In the presence of SmI_2_ and thiols **83**, **93**, **111**, and **150**, 5-oxotridecanal **189** was converted to lactone **190** (Scheme 34) [12]. The Lewis acid (R*S)SmI_2_ can promote the addition of R*SH to the aldehyde group of compound **189**. The samarium-bound hemithioacetal intermediate (A) can then undergo an intramolecular hydride shift to form the δ-hydroxy acid thioester intermediate (B). The reaction is capable of proceeding further with irreversible lactonization, releasing the catalyst (R*S)SmI_2_ for the next cycle. The presented examples clearly show that hydroxythiols more stereoselectively co-catalyze the lactonization of ketoaldehyde **189**.

## 5. Conclusions

In summary, the synthesis of acyclic, mono-, and bicyclic monoterpene thiols has been achieved via numerous pathways. The current review outlines a wide range of reactions to demonstrate the synthetic importance of functionalized monoterpenoids. In addition to focusing on the synthesis of monoterpene thiols, this review also examines their use as convenient and versatile synthons in organic synthesis and for the production of bioactive compounds.

## Data Availability

Data are available in the manuscript.
